# RETRACTED ARTICLE: “*Mycobacterium mephinesia*”, a *Mycobacterium terrae* complex species of clinical interest isolated in French Polynesia

**DOI:** 10.1038/s41598-019-47674-8

**Published:** 2019-08-01

**Authors:** Jamal Saad, Michael Phelippeau, May Khoder, Marc Lévy, Didier Musso, Michel Drancourt

**Affiliations:** 1 Aix-Marseille Univ., IRD, MEPHI, IHU Méditerranée Infection, Marseille, France; 2 Cabinet de Pneumologie de Taravao, Tahiti, Polynésie française France; 3 Laboratoire de Bactériologie, Centre Hospitalier de la Polynésie française, Pirae, Tahiti, Polynésie française France; 4 Aix-Marseille Univ., IRD, VITROME, Institut Louis Malardé, IHU Méditerranée Infection, Marseille, France

**Keywords:** Respiratory tract diseases, Infectious diseases

Retraction of: *Scientific Reports* 10.1038/s41598-019-47674-8, published online 01 August 2019

The Editors have retracted this article.

After publication, concerns about participant consent were brought to the attention of the Editors. In response to an enquiry from the Editors the Authors have stated that, although the patient did provide consent for the use of this sample in research as is stated in the article, they did not provide written consent for the publication of a case report describing their treatment. The content of this article has therefore been removed to protect patient confidentiality.

Michel Drancourt disagrees with this retraction. None of the other Authors responded to the Editors’ correspondence about the retraction.

